# Diagnostic and prognostic value of deregulated long non-coding RNA RPPH1 in patients with severe community-acquired pneumonia: a retrospective cohort study

**DOI:** 10.1186/s12890-023-02507-3

**Published:** 2023-06-09

**Authors:** Pengtao Su, Pengbo Hu, Ling Xu, Bing Zhang

**Affiliations:** 1 Department of Emergency, Binzhou Medical University Hospital, No.661 Huanghe 2nd Road, Binzhou, 256600 China; 2 Department of Respiratory Critical Care Medicine, Binzhou Medical University Hospital, Binzhou, 256600 China

**Keywords:** Severe pneumonia, Mild pneumonia, Onset, Development, Severity, Outcomes

## Abstract

**Background:**

Severe community-acquired pneumonia (SCAP) is one of the most common critical and acute diseases in the respiratory and acute medicine department. The expression and significance of lncRNA RPPH1 (RPPH1) in SCAP were assessed aiming to explore a biomarker assisting in the screening and management of SCAP.

**Methods:**

This study is a retrospective study enrolled 97 SCAP patients, 102 mild community-acquired pneumonia (MCAP) patients, and 65 healthy individuals. The serum expression of RPPH1 of study subjects was evaluated using PCR. The diagnostic and prognostic significance of RPPH1 in SCAP was evaluated by ROC and Cox analyses. Meanwhile, the correlation of RPPH1 with patients’ clinicopathological features was evaluated by spearman correlation analysis to evaluate its role in assessing disease severity.

**Results:**

A significant downregulation of RPPH1 was observed in the serum of SCAP patients compared with MCAP and healthy individuals. RPPH1 was positively correlated with ALB (*r* = 0.74) and negatively correlated with C-reactive protein (*r* = -0.69), neutrophil-to-lymphocyte ratio (*r* = -0.88), procalcitonin (*r* = -0.74), and neutrophil (*r* = -0.84) of SCAP patients, which are associated with the development and severity of SCAP. Additionally, reduced RPPH1 was closely associated with the 28-day development-free survival of SCAP patients and served as an adverse prognostic indicator together with procalcitonin.

**Conclusions:**

Downregulated RPPH1 in SCAP could act as a diagnostic biomarker screening SCAP from healthy and MCAP individuals and act as a prognostic biomarker predicting patients’ disease conditions and outcomes. The demonstrated significance of RPPH1 in SCAP could assist the clinical antibiotic therapies of SCAP patients.

## Background

Community-acquired pneumonia (CAP) is an acute infection of the alveoli induced by various microorganisms [1]. Benefiting from the development of antibiotics, the infection of some bacteria, such as *Streptococcus pneumoniae*, has been suppressed, but the infection of atypical pathogenic bacteria represented by *Mycoplasma pneumoniae* was found to increase gradually due to the abuse of antibiotics [2, 3]. Severe community-acquired pneumonia (SCAP) is one of the most common critical and acute diseases in the respiratory and acute medicine department. Due the rapid development, SCAP showed a high mortality rate and has attracted great importance. There were no unified diagnostic criteria to discriminate SCAP from mild community-acquired pneumonia (MCAP), and the present criteria are based on clinical symptoms and external pulmonary signs [4]. However, these indicators auxiliary examinations in the onset and course of SCAP, which play a weak role in the early warning and lead to the delayed diagnosis [5, 6]. Therefore, exploring repaid, efficient, and sensitive biomarkers for SCAP would benefit the development of clinical work and the improvement of patients’ prognosis.

Blood is the most common clinical hematological specimen with simple operation and low cost, and blood routine examination has been widely carried out in primary hospitals. Currently, the identification of serum and plasma biomarkers for human diseases has become a novel research direction. Biomarkers could help the diagnostic classification, determine the duration of antibiotic therapy, and predict patients’ outcomes [7]. The clinical significance of non-coding RNAs, including circular non-coding RNAs (circRNAs), long non-coding RNA (lncRNA), and microRNA (miRNAs) in the screening and monitoring of human diseases has been noted. A previous study screened a series of abnormally expressed lncRNAs in severe pneumonia compared with mild pneumonia and healthy individuals, and established an lncRNA-target gene network [8]. LncRNA RPPH1 (RPPH1) was found to be downregulated in severe pneumonia. The dysregulation of RPPH1 in other diseases was reported to regulate disease development and predicts patients’ outcomes. For example, upregulation of RPPH1 in gastric cancer predicted the poor prognosis of patients and promoted cell proliferation and migration [9]. In colorectal cancer, RPPH1 was also demonstrated to facilitate cancer development and metastasis [10]. Therefore, the dysregulation of RPPH1 in severe pneumonia was also speculated to serve as a biomarker of SCAP for its early detection and prognosis prediction.

In this study, the expression of RPPH1 in SCAP, MCAP, and healthy individuals was compared to confirm its dysregulation. The potential of RPPH1 differential expression in discriminating SCAP patients and indicating patients’ prognosis was evaluated.

## Materials and methods

### Study subjects

This study is a retrospective study and had been approved by the Ethics Committee of Binzhou Medical University Hospital. There were 97 SCAP patients, 102 MCAP patients, and 65 healthy individuals included from 2019–2021. The inclusion and exclusion criteria of each group were as follows:

Both SCAP and MCAP patients were enrolled from the department of emergency and were diagnosed according to the guidance for diagnosis and treatment of community-acquired pneumonia in adults based on imaging results and clinical symptoms of fever, cough, shortness of breath, dyspnea, and pulmonary fixation of moderate and fine moist rales [11]. The discriminating criteria for SCAP and MCAP were: 1) dyspnea; 2) cyanosis; 3) multiple lobes involved or more than 1/3 of the lungs invaded; 4) pleural effusion; 5) extrapulmonary complications [12, 13]. Patients with any one of the above terms were diagnosed with SCAP. Patients with other infectious diseases, systemic diseases, immune diseases, or medical history of immunosuppressant or modulator were excluded.

The healthy individuals were the volunteers who received a physical examination at the same time in the physical examination center of Binzhou Medical University Hospital as SCAP and MCAP patients. All participants or their families had signed the informed consent. The demographic features, including age, gender, and medical history were collected and compared among groups to ensure the matched characteristics.

### Sample collection and indicator analyses

Fasting blood samples were collected within 24 h of admission or physical examination (for healthy individuals) into an anticoagulant tube and were centrifugated at 3000 r/min for 20 min to obtain the serum samples. Collected samples were stored at -80 °C until the following analyses.

Procalcitonin (PCT) and neutrophil gelatinase-associated lipocalin (NGAL) were assessed by fluorescence immunoassay method, D-dimer (D-D) was measured by quantified immunofluorescence method, C-reactive protein (CRP) was analyzed with immunoscattering turbidimetry method, and an automatic blood analyzer (Mindray, China) was employed for routine analysis of blood.

### RNA extraction and Real-time quantitative PCR

RNA was extracted from serum with the miRNeasy Serum Kit (Qiagen, USA) according to the manufacturer's protocols. The concentration and purity of extracted RNA were evaluated by Nano Drop2000 (Thermo Scientific, USA) based on the value of OD260/280.

RNA was reverse transcribed into cDNA with the RevertAid First Strand cDNA Synthesis Kit (Thermo Scientific, USA). cDNA was amplified with the SYBR Green PCR Kit (Qiagen, USA). Relative expression was calculated with the 2^−ΔΔCt^ method normalized to GAPDH.

### Follow-up

SCAP patients were followed up for 28 d to obtain their development-free survival by telephone or outpatient review. The endpoints were defined as disease development and related death. The follow-up data were analyzed by Kaplan–Meier followed by the log rank post-hoc test and Cox regression analyses.

### Statistical analyses

Experimental data were represented as mean ± SD and analyzed using SPSS 26.0 software (IBM, USA). Difference evaluation was performed with one-way ANOVA and Chi-square test with the average RPPH1 levels as the cutoff dividing patients into low-RPPH1 and high-RPPH1 groups (*P* < 0.05). The potential of RPPH1 in differentiating SCAP was assessed with ROC analysis, while the correlation of RPPH1 with patients’ clinicopathological features was evaluated by Spearman correlation analysis. Multivariate Cox regression analysis was performed to evaluate the prognostic significance of RPPH1 in SCAP.

## Results

### Basic information of study subjects

The age and gender composition of the three groups were matched with insignificant differences. The medical history mainly presented in hypertension, diabetes, and coronary heart disease (CHD), which also showed no significant differences. Compared with healthy individuals, SCAP, and MCAP patients showed a higher level of white blood cell (WBC), CRP, neutrophil-to-lymphocyte ratio (NLR), PCT, D-D, NGAL, and neutrophil (NEU) and a lower ALB (Table 1). The levels of CRP, serum albumin (ALB), NLR, PCT, and NEU of SCAP patients were significantly different from those of MCAP patients (Table 1).

**Table 1:** Basic characteristics of study subjects

	SCAP	MCAP	Healthy
Age (years)	66.84 ± 11.20 (41–89)	65.64 ± 9.52 (42–89)	66.80 ± 9.53 (41–83)
Sex	62/35	64/38	34/31
WBC (× 10^9^/L)	11.08 ± 2.46^*^	11.19 ± 3.41^*^	7.32 ± 1.49
CRP (mg/L)	36.29 ± 5.89^***#^	19.08 ± 5.61^**^	3.35 ± 1.59
ALB (g/L)	27.57 ± 3.68^**#^	32.18 ± 4.36^*^	39.23 ± 6.11
NLR	11.70 ± 5.83^***#^	8.37 ± 2.30^**^	1.47 ± 0.70
PCT (ng/mL)	1.80 ± 0.50^***#^	0.46 ± 0.19^**^	0.03 ± 0.01
D-D (ng/mL)	1817.18 ± 309.06^***^	1589.99 ± 264.19^***^	175.21 ± 35.24
NGAL (ng/mL)	326.36 ± 50.53^***^	256.13 ± 41.97^***^	4.75 ± 1.34
NEU (× 10^9^/L)	4.98 ± 1.72^**#^	3.23 ± 1.06^*^	2.20 ± 0.81
Medical history (n, %)			
Hypertension	46, 47.42	49, 48.04	28, 43.08
Diabetes	13, 13.40	14, 13.73	7, 10.77
CHD	23, 23.71	25, 24.51	14, 21.54

*WBC* white blood cell, *CRP* C-reactive protein, *ALB* serum albumin, *NLR* the neutrophil-to-lymphocyte ratio, *PCT* procalcitonin, *D-D* D-dimer, *NGAL* neutrophil gelatinase-associated lipocalin, *NEU* neutrophil, *CHD* coronary heart disease. ^*^P < 0.05, ^**^P < 0.01, ^***^P < 0.001 relative to healthy individuals; ^#^*P* < 0.05, ^##^*P* < 0.01 relative to MCAP

### Expression and diagnostic significance of RPPH1 in SCAP patients

**Fig. 1:**
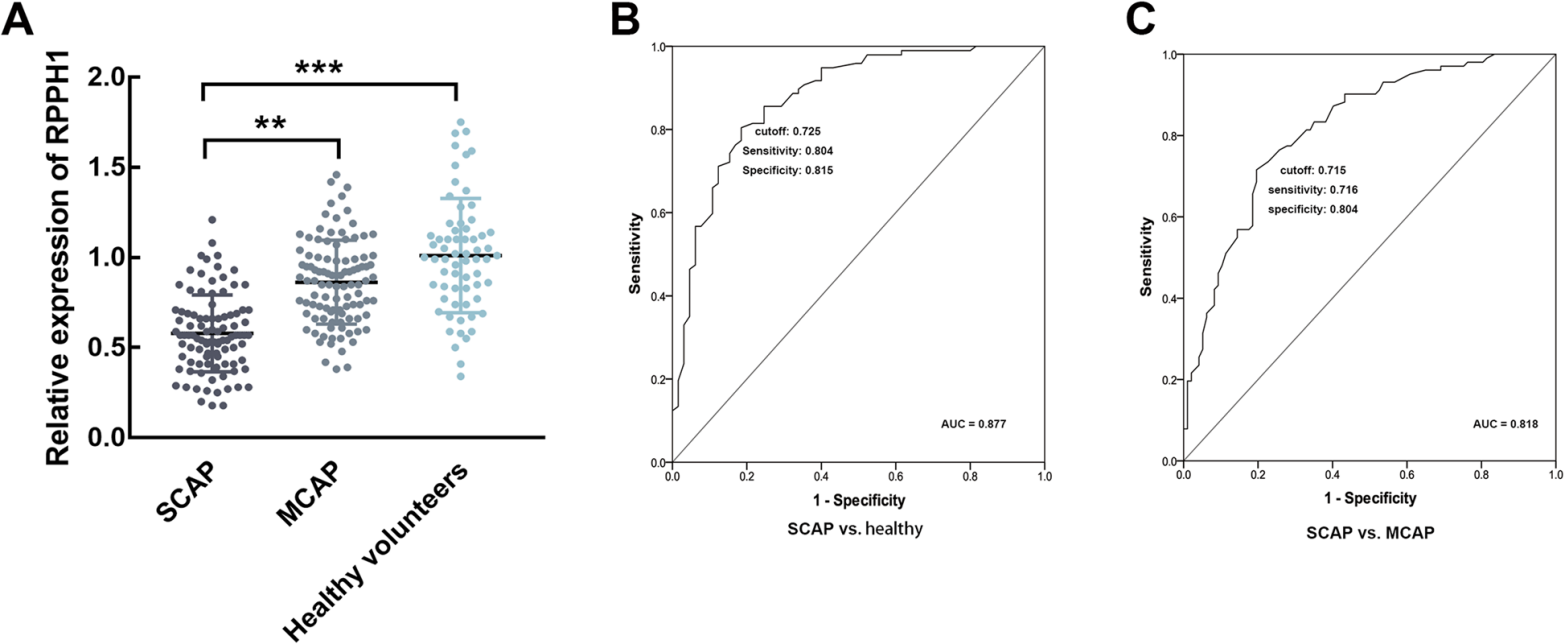
Expression and significance of RPPH1 in SCAP. **A** RRPH1 was downregulated in SCAP patients compared with MCAP and healthy individuals. **B**-**C** The downregulation of RPPH1 could discriminate SCAP patients from healthy (**A**) and MCAP (**B**) individuals with relatively high sensitivity and specificity. ^**^P < 0.01, ^***^P < 0.001

In the serum of SCAP and MCAP patients, RPPH1 was found to be significantly downregulated, while the expression of RPPH1 in SCAP was dramatically lower than that in the MCAP patients (Fig. 1A). The ROC results showed that the downregulated RPPH1 could discriminate SCAP patients from healthy individuals (AUC = 0.877, sensitivity = 0.804, specificity = 0.815, Fig. 1B) and MCAP patients (AUC = 0.818, sensitivity = 0.716, specificity = 0.804, Fig. 1C) with the cutoff of 0.725 and 0.715, respectively. The significance in differentiating SCAP from MCAP was relatively weak than that in distinguishing from healthy individuals.

### Association of RPPH1 with SCAP patients’ clinical features

**Fig. 2:**
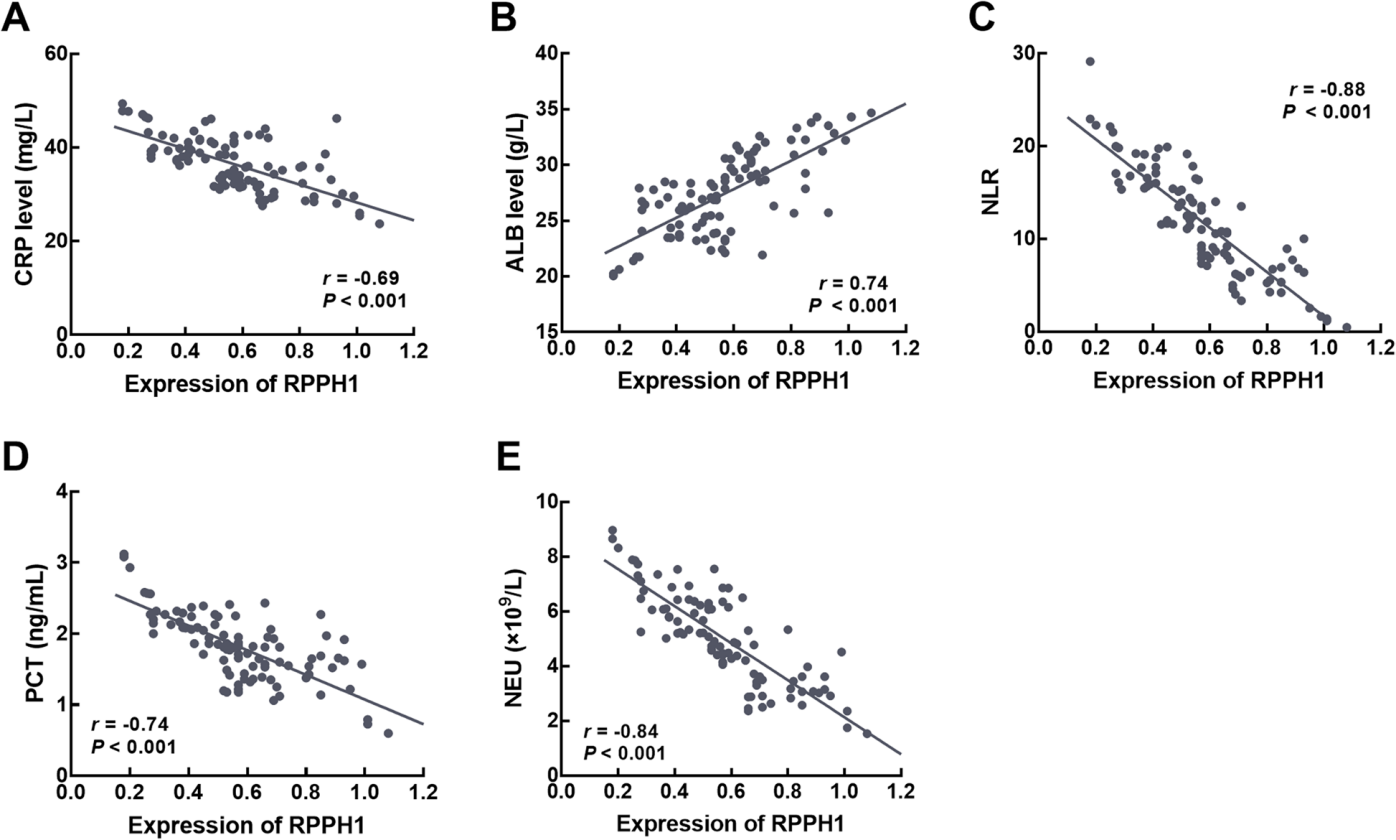
Correlation of RPPH1 with CRP (**A**), ALB (**B**), BLR (**C**), PCT (**D**), and NEU (**E**). A negative correlation was observed between RPPH1 and CRP (*r* = -0.69), NLR (*r* = -0.88), PCT (*r* = -0.74), and NEU (*r* = -0.84), while a positive correlation was observed between RPPH1 and ALB (*r* = 0.74)

For the basic clinicopathological characteristics of SCAP patients, the expression of RPPH1 was significantly correlated with CRP (*r* = -0.69, Fig. 2A), ALB (*r* = 0.74, Fig. 2B), NLR (*r* = -0.88, Fig. 2C), PCT (*r* = -0.74, Fig. 2D), and NEU (*r* = -0.84, Fig. 2E) of SCAP patients.

### Prognostic significance of RPPH1 in SCAP patients

**Fig. 3:**
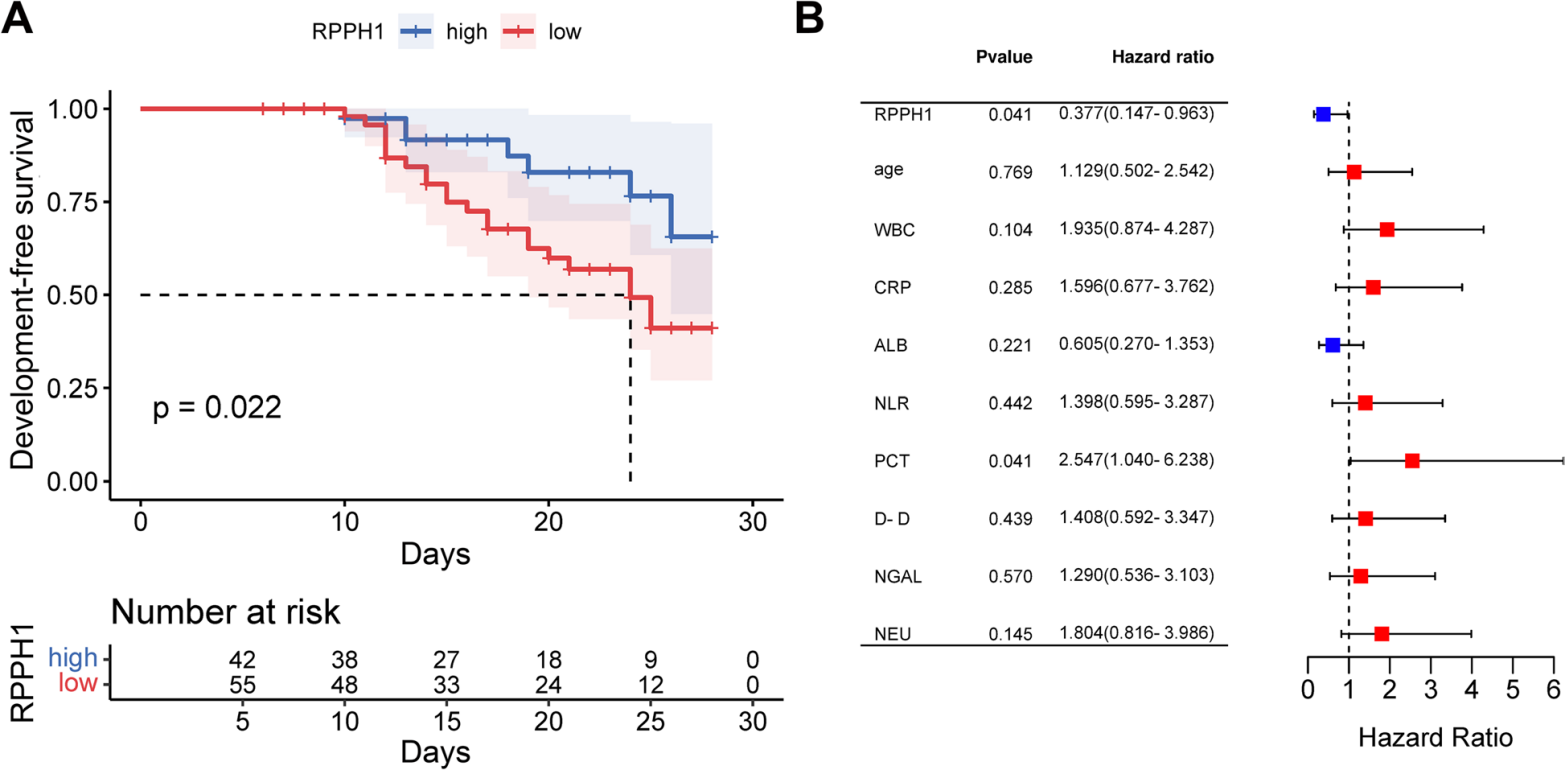
Prognostic significance of RPPH1 in SCAP. Lower expression of RPPH1 was significantly associated with the 28-day development-free survival of SCAP patients (**A**) and served as an prognostic factor together with PCT (**B**)

Patients were grouped according to the average serum expression of RPPH1 in SCAP patients. Patients with lower expression of RPPH1 showed a poorer 28-day development-free survival than the patients with higher RPPH1 expression (Fig. 3A). Additionally, RPPH1 (95% CI = 0.147–0.963) and PCT (95% CI = 1.040–6.238) were identified as prognostic factors of SCAP patients with the hazard ratio of 0.377 and 2.547, respectively (Fig. 3B).

## Discussion

SCAP is a global disease with high incidence and is the major reason for the death of global patients with pneumonitis. There was a lack of unified diagnosis criteria for SCAP, especially for the discrimination between SCAP and MCAP. In a recent study focused on children CAP, WBC, absolute neutrophil count, CRP and PCT were identified as effective biomarkers for the evaluation of disease severity [14]. However, the judgment of clinical symptoms is largely related to the evaluation bias of different clinical workers [15]. Although SCAP patients showed significant changes in several clinicopathological features, including CRP, ALB, NLR, PCT, and NEU, these characteristics are not stable and lacked the consideration of SCAP development. Recent studies have noted the clinical significance of RPPH1 in human diseases. In colorectal cancer, RPPH1 was revealed to boost tumor metastasis by promoting macrophage polarization and therefore associated with poor prognosis [10]. RPPH1 could also alleviated amyloid-β induced neuronal injury via sponging miR-122 [16]. For its clinical significance, RPPH1 was reported to indicate poor prognosis of gastric cancer patients and promotes the development of non-small cell lung cancer [9, 17]..

Serum samples are commonly studied specimens for exploring biomarkers. Herein, a significant downregulation of RPPH1 was observed in the serum of SCAP patients relative to both MCAP patients and healthy individuals. RPPH1 was also found to be significantly correlated with the CRP, ALB, NLR, PCT, and NEU, which were dramatically different between SCAP and MCAP. Although these cannot serve as diagnostic indicators of SCAP, these features are correlated with the severity and progression of SCAP. Serum CRP is sensitive to infection and inflammation and was revealed to be correlated with the survival of pneumonia patients that received antibiotic therapy and to predict the prognosis of CAP [18, 19]. ALB has also been identified as an independent indicator in the clinic [20]. Moreover, the CRP/ALB ratio was reported to benefit the early detection of SCAP and assess patients’ outcomes [21]. NLR could represent the balance between NEU and lymphocyte, which could indicate the severity of inflammation and infection. In previous studies, NLR was reported to predict the disease conditions and prognosis of adult CAP patients [22, 23]. Serum PCT was also identified as a prognostic biomarker of CAP, which was more significant than WBC and CRP [24]. The level of PCT could provide a reference for the time of antibiotic medication and withdrawal and guide the medication dosage [25]. The close association of RPPH1 with these indicators suggested that RPPH1 could indicate disease severity of SCAP and might be involved in the disease development. On the other hand, RPPH1 was also correlated with SCAP patients’ 28-day development-free survival. RPPH1 was identified as an prognostic factor of SCAP together with PCT. Therefore, RPPH1 served as a diagnostic and prognostic biomarker of SCAP providing a novel auxiliary indicator for the early detection and development evaluation of SCAP patients. It is regretful that MCAP patients have not been followed up to evaluate their disease development in the present study. If the changes in RPPH1 expression can be monitored during the period of MCAP patients converted to SCAP, it would further disclose the significance of RPPH1 in predicting the risk of SCAP occurrence and MCAP development. Hence, future investigations should prolong the follow-up time and also pay attention to the dynamic state of RPPH1 expression to better complete its clinical significance in human disease progression.

However, the present study is a single-center and retrospective study with a relatively small sample size. Some clinicopathological features of SCAP patients, such as CRP, ALB, NLR, and NGAL, were reported to possess significant diagnostic or prognostic value in CAP or other types of pneumonia, but their significance was insignificant in the present study, which might result from the small sample size. There have been various methods for the calculation and approximation of sample size. Hence, further studies should expand sample size and consider its rationality base on these methods [26–28]. Additionally, the pathogenesis of SCAP is various including the infection of different pathogenic bacteria [29–32]. The association of RPPH1 with pathogenic bacteria infection would improve the diagnostic efficiency and benefit the remedy for SCAP. Antibiotics are essential treatments for SCAP patients, of which the dosage and medication duration are critical factors determining therapeutic efficiency. The demonstrated significance of RPPH1 in SCAP could also provide guidance for the clinical antibiotic therapies of SCAP patients [33–35].

## Conclusions

In conclusion, downregulated RPPH1 in SCAP could act as a diagnostic biomarker screening SCAP from healthy and MCAP individuals and act as a prognostic biomarker predicting patients’ disease conditions and outcomes.

## Data Availability

The datasets used and/or analysed during the current study are available from the corresponding author on reasonable request.
